# Pleuro-Pneumonia Contagiosa

**Published:** 1883-07

**Authors:** 


### XIV.—PLEURO-PNEUMONIA CONTAGIOSA.

REPORTED FROM OBSERVATIONS KINDLY AFFORDED BY DR. HAWK.

The class went to Newark, on January 20th to investigate some typical cases of this disease prior to their destruction. We went from the station to where the animals were confined.

The previous history of the cases was as follows : They were native breed and were isolated from a herd of fourteen. Two animals prior to this case had contracted the disease and been destroyed. The one in particular which we had been called to see had contracted the disease so far as could be determined, eight days before. During that time she had lost in weight 150 pounds. Prior to the commencement of the attack she was considered in prime condition, and was then giving twelve quarts of good, rich milk.

From the onset of the attack the quantity of milk has gradually and steadily diminished and now is entirely suspended.

Although the milk has ceased flowing and there has been a marked loss of flesh, the general appearance of the animal is somewhat better than it has been.

At the period of invasion the hairs all pointed toward the head and the animal was very dull; had the dry cough peculiar to the disease; the back was arched; marked elevation of temperature; loss of appetite; bowels constipated; horns alternately hot and cold; breathing rough and harsh.

*Physical Examination.*—Revealed dullness over the whole left lung.

At the time seen by class the symptoms were as follows : Animal emaciated; stairing coat; back slightly arched; anterior feet wide apart when standing; posterior extremities well forward under the body; extremities cold; inspiratory and expiratory murmur irregular and harsh; no secretion of milk; mucous membranes pale. Respiration, 21 per minute.

*On Palpation.*—When the chest wall was manipulated there was marked evidence of tendernes.

The temperature at this time was 140° F., pulse 82, rapid and very feeble and small. No positive friction fremitus could be detected.

Upon auscultation bronchial breathing was the characteristic sound heard with a few crepitant rules.

The animal was immediately destroyed and a necropsy made.

The lungs were first removed. The right was firmly adherent to the chest wall and was about twice the normal weight.

The lungs were sent to the pathological labaratory for examination.

The specimen was the subject of one of the pathological lectures. The specimen showed all the four stages of this disease excepting the last, or that of gangrene. A small portion of the lung was in the primary stage or that of congestion; a small territory also was in the stage of red hepatization, but the greater portion was in the third stage or that of gray hepatization, which was rapidly passing to the suppurative and gangrenous condition.

The marbilization of the lung was well marked both by these different stages and the thickened bands separating the separate lobules. These bands between the lobules were of an inflammatory nature and were composed of several fibres, pus and a little blood. Each little lobule is a separate lung in miniature. In the cow's lung, being so large and distinctly separated, they can, in the normal state, be readily recognized, but now they are still plain. In the human being the lobule are so small and so closely packed together it is almost impossible to isolate them. This last is also true of the horse's lung. There was also present, as a marked lesion, a bronchitis, but this, in our belief, is a secondary process to the pleurisy and interstitial pneumonia.

